# Management and outcome of hypertrophic obstructive cardiomyopathy in pregnant women: a case report

**DOI:** 10.11604/pamj.2021.38.140.25699

**Published:** 2021-02-08

**Authors:** Maryem Assamti, Ramia Bougrine, Nabila Ismaili, Noha El Ouafi

**Affiliations:** 1 Department of Cardiology, Mohamed VI University Hospital, Mohamed First University of Oujda, Oujda, Morocco,; 2 Laboratory of Epidemiology and Clinical Research, Mohamed First University of Oujda, Oujda, Morocco

**Keywords:** Hypertrophic cardiomyopathy, pregnancy, sudden cardiac death, case report

## Abstract

Hypertrophic cardiomyopathy (HCM) is a common inherited cardiomyopathy, with an estimated prevalence of 1 in 500 people. Despite overall favorable outcomes with modern treatment and early diagnosis of disease, adverse complications could occur during times of physiological stress like pregnancy. Complications of HCM include sudden cardiac death, heart failure, and arrhythmia. We report the case of a 32-year-old pregnant woman with obstructive HCM, presenting with recurrent episodes of ventricular arrhythmia despite medical therapy. This case exhibits how close monitoring and proper management during pregnancy according to the latest recommendations, resulted in a successful and uneventful delivery.

## Introduction

Hypertrophic cardiomyopathy (HCM) is a genetic condition characterized by primary left ventricular hypertrophy that is not related to abnormal loading conditions (such as hypertension or valvular disease) [1]. This disease is often caused by mutations within genes encoding cardiac sarcomeric proteins [2]. Although pregnancy is well tolerated by HCM patients [3]; related hemodynamic burdens may lead to unfavorable events requiring close monitoring and adequate treatment.

## Patient and observation

A 32-year-old pregnant woman presented at 36 weeks gestation for further management of known HCM. She was recently diagnosed with HCM at 22 weeks of gestation, after she presented with paroxysmal palpitations. She had a history of a sudden cardiac death of her sister at the age of 15, and one of her brothers at the age of 11. Her first pregnancy was well tolerated. Her first echocardiogram showed a mild left ventricular outflow tract obstruction with a gradient at 30 mmHg, and a septum thickness of 15 mm. An ambulatory electrocardiogram monitoring showed isolated monomorphic ventricular extrasystoles, without ventricular tachycardia. Her SD score was then at 4%, an implantable cardioverter defibrillator (ICD) was proposed but the patient refused it; she was treated with bisoprolol only.

**Figure 1:**
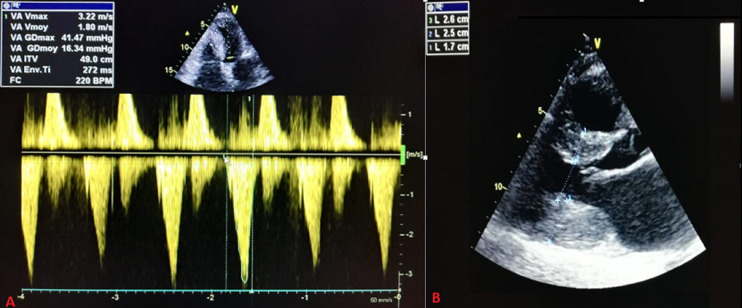
transthoracic echocardiography showing: A) a max instantaneous late peak gradient of 42 mmHg measured with continuous wave (CW) Doppler; B) a posterior wall thickness at 26 mm and a septum thickness at 17 mm measured on a parasternal long axis view

At 36 weeks of gestation, she consulted in our hospital. She reported palpitations; without dyspnea, nor chest pain, or syncope. Her physical examination revealed a blood pressure (BP) of 110/65 mmHg, a regular pulse rate of 108 beats per minute, anda mid-systolic murmur over the lower left sternal border.The electrocardiogram showed sinus rhythm. An echocardiogram demonstrated a posterior wall thickness at 26 mm, a septum thickness at 17 mm; and a mild increase in LVOT obstruction with agradient of 42 mmHg at rest; without an increase at standing up or Valsalva maneuvers; nor systolic anterior motion of the mitral valve, and a restrictive mitral profile (Figure 1). A 48 hours electrocardiography (ECG) monitoring revealed multiple short runs of non-sustainedventricular tachycardia. Pro BNP and BNP dosage were unavailable. The reassessed sudden cardiac death risk score was higher at 14%.

After a comprehensive discussion among cardiologists, gynecologists, and anesthesiologists, we decided to perform a vaginal delivery. At 37 weeks of gestation, the patient was admitted to the obstetric department, and an induction of labor was initiated under close maternal and fetal monitoring with maintained medical therapy. The patient gave birth to a healthy female newborn after an uneventful delivery. The patient remained on beta-blockers, and she is currently asymptomatic.

## Discussion

Hypertrophic cardiomyopathy (HCM) is the most common genetic cardiac disease, with an estimated prevalence of 0.2% in the general population [4], and less than 0.1% in childbearing women [3]. Hypertrophic cardiomyopathy is associated with an increased risk of sudden cardiac death, arrhythmia, and heart failure. The incidence of complications varies between 5 and 48%, depending on the size of the samples and the study design. While the maternal mortality is estimated at 0.5% [3]. According to the ROPAC registry, which prospectively included 60 pregnant women with HCM, 23% of the patients developed important MACE (including HF and arrhythmias). They observed that NYHA functional class > 2 and signs of heart failure before pregnancy were significantly associated with cardiovascular events. Surprisingly, no significant differences in pregnancy outcome were found between women with obstructive and non-obstructive HCM [5]. Autore *et al*. also noticed that clinical deterioration occurred more frequently in women with LV outflow tract obstruction, without statistically significant difference [6]. In contrast, in a retrospective review of 27 pregnancies in 23 women with HCM, Tanaka *et al*. reported a cardiovascular event rate of 48%; the vast majority involved arrhythmia and occurred during the 3^rd^ trimester. They also identified the use of cardiac medication prior to pregnancy as a predictor of maternal cardiac events [7]. This condition may influence the fetal outcome. Schinkel reported in his literature review a 15% rate of spontaneous abortion and a 2% stillbirth rate, which is comparable to the general population. However, premature birth was more frequent, and was observed 26% of cases [8].

Therefore, an appropriate assessment antepartum and proper monitoring and medical management are crucial to avoid related morbidity. In general, the diagnosis of HCM is confirmed when left ventricular (LV) myocardial thickness exceeds 15 mm on echocardiography, cardiac magnetic resonance (CMR) imaging, or computed tomography (CT) [9]. Once the diagnosis is confirmed, clinical status, left ventricular outflow tract (LVOT) obstruction, ventricular function, and arrhythmias should be assessed. Besides, establishing baseline levels of serum BNP or NT-proBNP, before or at the beginning of pregnancy, and throughout pregnancy in symptomatic patients, or at the onset of symptoms, can reflect hemodynamic deterioration [10]. Women in WHO class II(mild to moderate LVOT obstruction, asymptomatic with or without medication, well-controlled arrhythmia, normal systolic LV function, or mild LV dysfunction) should be assessed each trimester, and those in WHO class III (severe LVOT obstruction, symptoms or arrhythmias despite optimal medication, moderate systolic LV dysfunction) should be assessed monthly or bimonthly [1, 3].

In WHO class IV with severe symptomatic LVOT obstruction or severe systolic LV dysfunction, pregnancy is in principle contraindicated [1]. In order to prevent undesired events, and culminate in a smooth delivery, appropriate peripartum management and close monitoring are essential. According to the latest ESC guidelines, beta-blockers should be continued if they are already being taken, and should be started in case of new-onset of arrhythmia (preferably metoprolol) [3]. Complications such as neonatal hypoglycemia, bradycardia, and apnea should be anticipated [9]. Verapamil and disopyramide are a second choice treatment if beta-blockers are not tolerated [11]. Antiarrhythmic drugs should be administered with caution because of the potential fetotoxic effects [9]. Anticoagulation is recommended for paroxysmal or persistent AF, regardless of the CHADS VASC score. Low molecular weight heparin with anti-factor-Xa monitoring (peak anti-Xa level 0.8-1.2 U/mL 4-6 h post-dose) in the first trimester and from the 36^th^ week onwards, and VKAs are recommended in the second and third trimester. NOACs are not recommended because of proven toxicity in animals and insufficient data in humans [3]. Patients with a history or family history of sudden death (such as in our case) need close surveillance and prompt investigation if they present symptoms of palpitations or presyncope. When indicated, an ICD should be implanted preferably with echocardiographic guidance [3].

**Figure 2:**
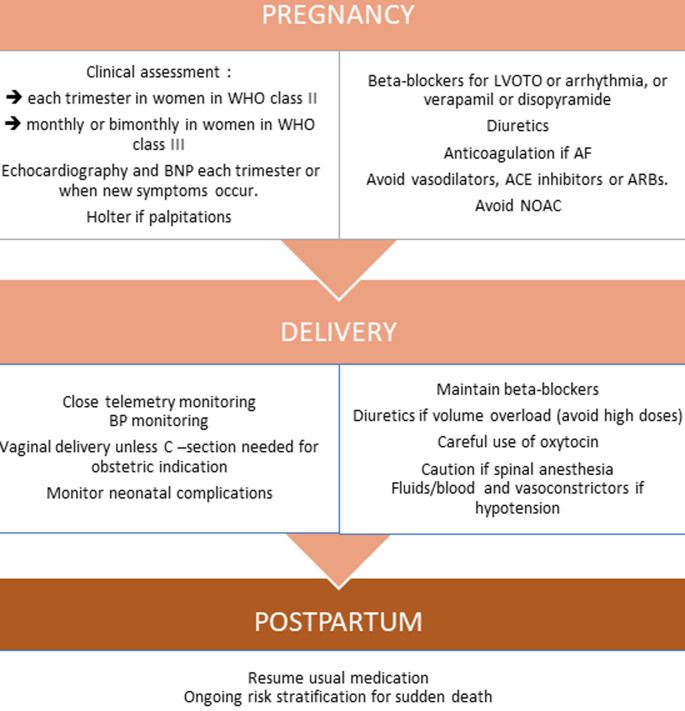
proposed algorithm for assessment and management of HCM during pregnancy

In order to reduce the outflow gradient, alcohol septal ablation has been described. There are only two recent case reports of alcohol septal ablation during pregnancy, but only one of them was successful [12]. This suggests that ASA could be performed, when conservative therapy is ineffective, but should ideally be considered before conception in high-risk patients. Regarding the mode of delivery, cesarean section should be considered mostly for obstetric indications, or in patients with severe left ventricular (LV) outflow tract obstruction, pre-term labor while on oral anticoagulation, or severe heart failure (HF) [3]. Tocolytic agents with ß-adrenergic stimulating activity should be avoided in women with obstructive HCM [9]. Oxytocin should be given as a slow infusion, because of vasodilatory properties. Epidural and spinal anesthesia must be used cautiously, especially with severe LV outflow tract obstruction, since it may induce hypotension. A close monitoring of heart rhythm should be considered in patients with a high risk of developing arrhythmias [3]. Despite the high risk of endocarditis associated with HCM, prophylactic antibiotic treatment before delivery is not recommended [13] (Figure 2).

## Conclusion

In conclusion, and despite the paucity of data, the complication rate in pregnant women with HCM appears to increase in case of a history of previous cardiac events, poor functional class, and LVOT obstruction. Therefore, early screening of this disease, identifying women at high risk of adverse outcomes and counseling on contraception, is essential in HCM women. Furthermore, it is critical to inform women with HCM regarding the risk of genetic transmission to their offspring.
